# Automatic Detection of Orientation Contrast Occurs at Early but Not Earliest Stages of Visual Cortical Processing in Humans

**DOI:** 10.3389/fnhum.2018.00369

**Published:** 2018-10-01

**Authors:** Yanfen Zhen, Duo Li, Ran Ding, Zili Huang, Zhe Qu, Yulong Ding

**Affiliations:** ^1^Department of Psychology, Sun Yat-sen University, Guangzhou, China; ^2^School of Psychology, South China Normal University, Guangzhou, China

**Keywords:** orientation contrast, automatic processing, top-down attention, C1, event-related potentials (ERPs)

## Abstract

Orientation contrast is formed when some elements orient differently from their surroundings. Although orientation contrast can be processed in the absence of top-down attention, the underlying neural mechanism for this automatic processing in humans is controversial. In particular, whether automatic detection of orientation contrast occurs at the initial feedforward stage in the primary visual cortex (i.e., V1) remains unclear. Here, we used event-related potentials (ERPs) to examine the automatic processing of orientation contrast in humans. In three experiments, participants completed a task at fixation while orientation contrasts were presented in the periphery, either in the upper visual field (UVF) or the lower visual field (LVF). All experiments showed significant positive potentials evoked by orientation contrasts over occipital areas within 100 ms after stimulus onset. These contrast effects occurred 10–20 ms later than the C1 components evoked by identically located abrupt onset stimuli which indexes the initial feedforward activity in V1. Compared with those in the UVF, orientation contrasts in the LVF evoked earlier and stronger activities, probably reflecting a LVF advantage in processing of orientation contrast. Even when orientation contrasts were rendered almost invisible by backward masking (in Experiment 2), the early contrast effect in the LVF was not disrupted. These findings imply that automatic processing of orientation contrast could occur at early visual cortical processing stages, but was slightly later than the initial feedforward processing in human V1; such automatic processing may involve either recurrent processing in V1 or feedforward processing in early extrastriate visual cortex.

**Highlights**

- We examined the earliest automatic processing of orientation contrast in humans with ERPs.
- Significant orientation contrast effect started within 100 ms in early visual areas.
- The earliest orientation contrast effect occurred later than the C1 evoked by abrupt onset stimuli.
- The earliest orientation contrast effect was independent of top-down attention and awareness.
- Automatic detection of orientation contrast arises slightly after the initial feedforward processing in V1.

## Introduction

Human beings are exposed to a large number of visual stimuli in daily life. Usually, only a small portion of these stimuli are subjected to top-down attention and consciously processed, while the others do not undergo sufficient processing (see Desimone and Duncan, 1995 for a review). However, some salient visual stimuli might be processed even without top-down attention (Braun and Sagi, 1990; Nothdurft, 1992, 1993; Theeuwes, 1992; Wolfe, 1992). This automatic processing, which occurs regardless of the viewer’s goals, helps to avoid missing important information for appropriate behavior, and is critical for the survival in the environment.

One common type of salient visual stimulus is abrupt onset stimulus. After the presence of an abrupt onset stimulus, the corresponding brain activation spreads rapidly from the primary visual cortex (i.e., V1) to other high-level cortical areas, which is termed a feedforward sweep (Lamme and Roelfsema, 2000). In single unit studies of animals, the earliest neural activity of V1 starts at about 40 ms after stimulus onset (Lamme et al., 1998b; Knierim and van Essen, 1992; Lee et al., 1998; Poort et al., 2012). In event-related potential (ERP) studies of humans, the initial feedforward processing in V1 is reflected by the earliest visual component C1 (Jeffreys and Axford, 1972; Di Russo et al., 2003), which starts at 50–60 ms after stimulus onset. At longer latencies, activities in low level cortical areas can be modulated by information from cells at the same level through horizontal connection, as well as information from higher levels through feedback connections (for reviews see Lamme et al., 1998a; Lamme and Roelfsema, 2000). A large number of ERP studies have found that top-down attention could not modulate the initial feedforward activity in V1, but could influence later cortical processing stages (Clark and Hillyard, 1996; Martínez et al., 1999; Di Russo et al., 2003; Ding et al., 2014; Baumgartner et al., 2018). These findings suggest that, for an abrupt onset stimulus, the initial feedforward processing in V1 is automatic and independent of top-down attention.

Visual stimuli could also be salient when there is local difference of a single elementary visual feature (e.g., orientation, motion direction and color) in a homogeneous background (Nothdurft, 1993, 2000; Zhaoping, 2008; Zhang et al., 2012). For example, horizontal bars embedded in a vertical bar array are salient due to the orientation difference, which produces an example of orientation contrast. As shown by previous single unit studies of monkeys, processing of orientation contrast could be reflected in the neuronal response in V1. That is, for a given line segment, the response in V1 neurons differed when it was surrounded by differently oriented relative to same oriented background elements (Knierim and van Essen, 1992; Sillito and Jones, 1996; Nothdurft et al., 1999; Jones et al., 2002). Such modulation has also been uncovered in studies of monkeys with orientation contrast in texture filled with randomly positioned line segments (Lamme, 1995; Zipser et al., 1996; Lee et al., 1998; Lamme et al., 1999; Supèr et al., 2001; Poort et al., 2012). In all these studies, neural response signaling an orientation contrast started about 20 ms (Knierim and van Essen, 1992; Lee et al., 1998; Nothdurft et al., 1999, 2000; Poort et al., 2012) or slightly longer (Lamme, 1995; Zipser et al., 1996; Supèr et al., 2001) after the initial response signaling stimulus onset in V1. Such delayed modulation was found in V1 neurons regardless of whether orientation contrast defined the target of a saccade (Lamme, 1995; Zipser et al., 1996; Lee et al., 1998; Lamme et al., 1999; Supèr et al., 2001; Poort et al., 2012), or was totally task-irrelevant (Lamme et al., 1998b; Knierim and van Essen, 1992; Nothdurft et al., 1999; Poort et al., 2012). These findings suggest that the processing of orientation contrast in monkeys can be automatic and starts slightly after the initial feedforward cortical processing in V1 (for reviews see Lamme et al., 1998a; Lamme and Roelfsema, 2000).

In human participants, however, more evidence is still needed to clarify the time course of orientation contrast processing. A number of studies have used ERP to examine the brain activities evoked by orientation contrasts embedded in a texture array. In most of these studies, ERP of an orientation contrast was defined as the difference ERP between a homogenous array and an otherwise-identical array containing an orientation contrast. To examine the automatic processing of orientation contrast, previous studies usually presented orientation contrasts as task-irrelevant stimuli and outside the focus of top-down attention. In humans, it remains an open question whether the initial feedforward processing stage in V1 is involved in the automatic processing of orientation contrast. For example, Scholte et al. (2008) found that a task-irrelevant orientation contrast did not evoked significant activity at occipital sites until 92 ms after stimulus onset. In some other studies, orientation contrasts evoked even later activities (Schubö et al., 2001; Guzzon and Casco, 2011). It is important to note that influence of orientation contrast on the C1 was not the focus of these studies and none of them elicited an evident C1 component. Since the polarity and amplitude of C1 are highly dependent on stimulus locations (Jeffreys and Axford, 1972; Clark et al., 1995), the C1 can be observed in scalp ERPs only by presenting stimuli in suitable locations. In the study of Schubö et al. (2001), the portions of texture array that contained orientation contrast were either on the left or right side of the fixation, which is not optimal for eliciting a C1 component. In other studies, the texture regions containing orientation contrasts were distributed across the upper visual field (UVF) and lower visual field (LVF), and early activities evoked by orientation contrasts in different locations might cancel out each other (Scholte et al., 2008; Guzzon and Casco, 2011). Recently, one study used texture stimuli suitable to elicit a C1 component and reported that orientation contrasts could modulate C1 amplitudes (Zhang et al., 2012). This finding is in line with the V1 saliency hypothesis (Li, 2002), which suggests that the most salient location is the spatial receptive field of the most responsive V1 cells. However, the orientation contrasts in Zhang et al. (2012) were always task-relevant and subjected to top-down attention. In such a situation, the activities related to bottom-up processing and those related to top-down attention were undistinguishable. Therefore, it remains unclear whether the automatic processing of orientation contrast in humans could occur as early as the C1.

Taken together, the early automatic processing of orientation contrast in humans needs to be revisited in a way that not only minimizes top-down influence, but also benefits the observation of earliest ERP components. The present study adopted the following Experiment settings to achieve this goal.

First, to examine the automatic processing of orientation contrast without top-down attention, orientation contrasts were presented in the periphery while participants completed a luminance detection task at fixation in all three experiments. In addition, in one Experiment (Experiment 2), backward masking was used shortly after the presentation of orientation contrasts to block participants’ awareness. In this case, we want to examine early orientation contrast effects in the absence of top-down attention as well as visual awareness.

**Figure 1:**
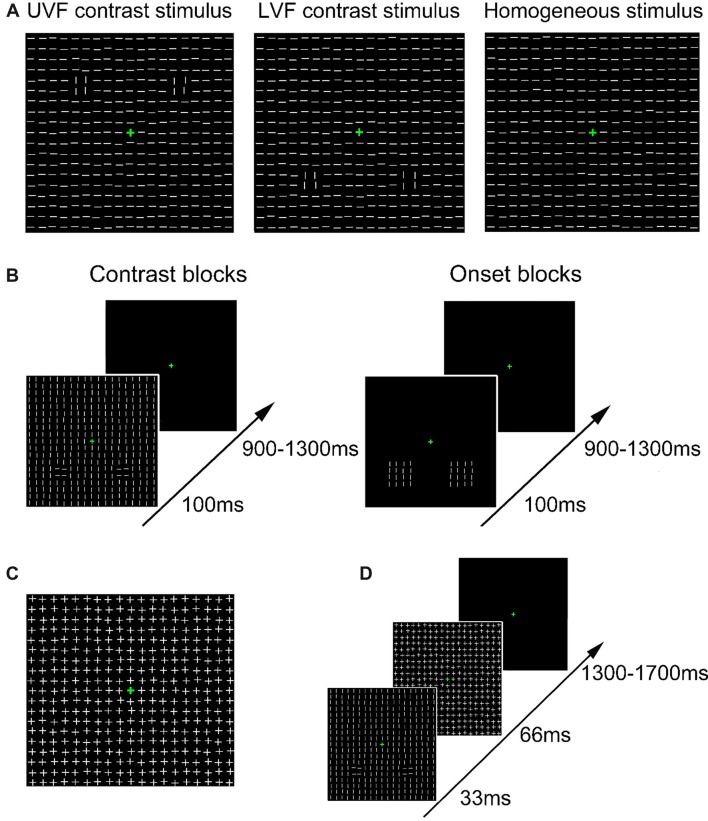
Schematic presentation of stimuli and trial procedure.** (A)** Schematic presentation of the task-irrelevant textures presented in Contrast blocks in Experiment 1. Heterogeneous texture stimuli were embedded with orientation contrasts in either the upper visual field (UVF; left) or the lower visual field (LVF; middle). Homogeneous texture consisted of only identically oriented line elements (right). For each type of stimulus, 50% were made of horizontal background elements (as shown in the figure), and the other 50% were made of vertical background elements. **(B)** Schematic presentation of the trial procedure in Contrast blocks (left) and Onset blocks (right) in Experiment 1. Participants performed a luminance detection task at fixation while texture arrays were presented as task-irrelevant stimuli. The orientations of all line segments were vertical or horizontal in Onset blocks in different trials. **(C)** Schematic presentation of the mask stimulus in Experiment 2. **(D)** Schematic presentation of the trial procedure in Experiment 2. Participants performed a same luminance detection task as in Experiment 1.

Second, suitable stimuli were adopted to benefit the observation of possible C1 effects. Similar to Zhang et al. (2012), the present study used big texture stimuli of line segments, and small regions of orientation contrasts were embedded either in the UVF or in the LVF for a given trial. In Experiment 1 and 2, instead of presenting the whole texture stimuli in only the UVF or LVF as Zhang et al. (2012) did, we used centrally symmetric texture stimuli that covered both the UVF and LVF (Figure 1) to facilitate participants’ fixation at the center. In Experiment 3, the whole texture stimuli were presented only in the UVF or LVF in a trial, while the locations of orientation contrasts were the same as in Experiment 1 and 2. As shown in previous animal studies, response of a V1 neuron can be significantly influenced by contextual input outside but near its receptive field (Knierim and van Essen, 1992; Lamme, 1995). These findings suggest that a contrast effect at early visual cortical processing stages is highly local. This idea agrees with the V1 saliency hypothesis (Li, 2002; Zhaoping, 2008), which proposed that saliency of a location is determined by the contrast between that location and its context. Thus, if there is any C1 contrast effect, its polarity and amplitude may mainly depend on where the orientation contrasts are located rather than where the overall texture stimuli are presented.

In addition, to ensure that the locations of orientation contrasts in the present study are suitable to evoke robust C1 components on the scalp, we also presented abrupt onset stimuli at the same locations in Experiment 1. We expect to find that abrupt onset stimuli, which share the locations with orientation contrasts, could elicit typical C1 components. If detection of orientation contrasts involves initial responses of V1, then the early contrast effects should resemble the C1 evoked by the abrupt onset stimuli in some key properties, such as polarity, latency and scalp distribution (Luck, 2005). However, if the early contrast effects show different properties from the C1 of the abrupt onset stimuli (for example, having longer latencies than the C1), we may infer that detection of orientation contrast occurs later than the initial feedforward processing in human V1.

## Experiment 1

### Method

#### Participants

Twenty-four healthy young volunteers (seven males, mean age = 20.6 years, range = 18–28 years) took part in Experiment 1. All participants had normal or corrected-to-normal vision and were right-handed. All volunteers were compensated for their participation with payment or with credit hours fulfilling a course requirement. Written informed consent was obtained from each participant before the experiment. This study was approved by the ethic committee of Sun Yat-sen University and was conducted in accord with the Code of Ethics of the World Medical Association (Declaration of Helsinki).

#### Stimuli and Task

In Experiment 1, two kinds of blocks (Contrast blocks and Onset blocks) were adopted to examine the ERPs evoked by orientation contrasts and abrupt onsets, respectively. As shown in Figure 1, two different sets of texture stimuli were presented in these two kinds of blocks, both of which consisted of white line segments (8.0 cd/m^2^, 0.76° × 0.1° each, spaced 1.16° apart) slightly “jittered” (0°–0.1°) on a black background (0.04 cd/m^2^).

In Contrast blocks, texture stimuli were either homogeneous or heterogeneous. Each texture stimulus consisted of a 19 × 19 array of line segments (21.7° × 21.7°, Figure 1A), with the center replaced by a green fixation cross. Homogeneous texture stimuli were composed of identically oriented line segments (Figure 1A, right). For heterogeneous texture stimuli, two regions of 2 × 2 line segments (1.92° × 1.92°) with an orthogonal orientation were embedded in the background in either the UVF (Figure 1A, left) or LVF (Figure 1A, middle). These two types of heterogeneous texture stimuli were called UVF contrast stimuli and LVF contrast stimuli, respectively. According to previous studies (Nothdurft, 1993, 2000; Zhaoping, 2008; Zhang et al., 2012), the orthogonal lines and their differently oriented surrounding background elements consisted of two orientation contrasts. The two orientation contrasts were presented bilaterally, with centers 7.4° from the fixation point. There were six Contrast blocks of 288 trials, resulting in a total of 1,728 trials. Homogeneous stimuli, UVF contrast stimuli and LVF contrast stimuli were presented randomly in a block with equal probabilities. For each type of stimulus, there were 576 trials, among which 50% were made of horizontal background elements, and the other 50% were made of vertical background elements. Stimuli with different background orientations were presented randomly and were pooled in the analysis, so that the difference between homogenous and heterogeneous stimuli was related to orientation contrast itself rather than specific orientations. The size and spacing of the line segments, as well as the positions of the orientation contrasts, were similar to those used by Zhang et al. (2012).

In Onset blocks, we examined ERPs evoked by abrupt onsets presented in same locations with the orientation contrasts in Contrast blocks. Since an orientation contrast in Contrast blocks was formed by both a 2 × 2 array and its surrounding elements, a 4 × 4 array (4.2° × 4.2°) was adopted for an onset stimulus to match the size of the orientation contrast. In each trial, a pair of onset stimuli was presented bilaterally in either the UVF or LVF (Figure 1B, right). All line segments were horizontal in half of the trials, and vertical in the other half. The centers of the onset stimuli were 7.4° from the fixation point. The center locations of these abrupt onset stimuli are identical to those of the orientation contrast regions in Contrast blocks. There were four Onset blocks of 288 trials, resulting in a total of 1,152 trials. The UVF onset stimuli and the LVF onset stimuli were presented randomly with equal probabilities. Thus, same to Contrast blocks, there were 576 trials for each type of stimulus.

Trial procedure was identical in Contrast blocks and Onset blocks (Figure 1B). In both kinds of blocks, participants were instructed to maintain their fixation at a green central cross (0.6° × 0.6°, 8.4 cd/m^2^, RGB: [0, 180, 0]) throughout the experiment. The luminance of the central cross increased for 100 ms every 3–6 s randomly, and participants were required to press a predefined key whenever they detected a luminance change. The target luminance in the first block was set to be 14.3 cd/m^2^ (RGB: [0, 230, 0]). Target luminance in the following blocks was adjusted according to participants’ performance to ensure that the overall hit rate was ~80%–90%. Specifically, if the hit rate was higher than 90% in a block, the target RGB value would decrease for [0 10 0] in the next block. If the hit rate fell below 80% in a block, the target RGB value would increase for [0 10 0] in the next block. Each task-irrelevant stimulus (contrast or onset stimulus) was presented for 100 ms, with a 900–1,300 ms interval between successive stimuli. The presentations of central targets and task-irrelevant stimuli were randomized independently to further avoid temporal attention and/or expectation of task-irrelevant stimuli (see Summerfield and Egner, 2009 for a review). To avoid possible spatial priming effects induced by abrupt onset stimuli around the orientation contrast regions, all participants first completed six Contrast blocks, and then four Onset blocks. Each block lasted about 6–7 min. To avoid fatigue effects, participants took a short break (10 s) every 48 trials, and a longer break (several minutes) after each block. The whole Experiment lasted for about 1.5 h, including participants’ breaks between blocks.

#### EEG Recordings

An ANT EEG/ERP acquisition system, with Refa-8 72-channel DC amplifier and ASA software, was used in electroencephalogram (EEG) recording. EEGLAB^1^ was used in offline EEG data processing. The scalp EEG was recorded from an array of 58 electrodes (including the following sites: FP1, FPZ, FP2, AF3, AFZ, AF4, F7, F3, FZ, F4, F8, FC5, FC3, FC1, FCZ, FC2, FC4, FC6, T7, C5, C3, C1, CZ, C2, C4, C6, T8, TP7, CP5, CP3, CP1, CPZ, CP2, CP4, CP6, TP8, P7, P5, P3, P1, Pz, P2, P4, P6, P8, PO7, PO3, POz, PO4, PO8, O1, Oz, O2, I5, I3, Iz, I4 and I6 from the 10/10 system). The horizontal and vertical electro-occulogram (EOG) was recorded as well. The EEG was recorded with a common average reference on-line, and was then algebraically re-referenced to the average of the left and right mastoid. Electrode impedance was kept to less than 5 kΩ. The EEG analog signal was digitized at a 512-Hz sampling rate, and a digital anti-aliasing filter of 0.27× sampling rate was applied at the time of recording. During the offline analysis, a Blackman windowed sinc finite impulse response (FIR) filter with a half-amplitude cut-off frequency of 40-Hz and transition bandwidth of 20-Hz was used for low-pass filtering, and a short infinite impulse response (IIR) filter with a half-amplitude cut-off frequency of 0.1-Hz and transition bandwidth of 0.084-Hz was used for high-pass filtering on the continuous EEG data. Then the epoch was extracted, including 200 ms of pre-stimulus and 600 ms of post-stimulus. The trials contaminated by eye blinks, eye movement or muscle potentials exceeding ±70 μv at any electrode were excluded before averaging, as were data surrounding a button press (−650 to +650 ms). After artifact rejection, about 400 trials were left for each type of stimulus (i.e., homogeneous stimulus, UVF contrast stimulus, LVF contrast stimulus, UVF onset stimulus, LVF onset stimulus). The baseline for ERP measurements was the mean voltage over the 100 ms pre-stimulus to stimulus onset, and the average waveforms were corrected by subtracting the mean voltage during this interval.

#### Statistical Analyses

For the central luminance detection task, hit rates and reaction times (RTs) for correct hit responses were calculated. Correct hits were defined as responses from 200 ms to 1,200 ms after target onsets. A pair-wise *t*-test was used to analyze the difference of Onset blocks and Contrast blocks in hit rates and RTs.

ERP mean amplitudes of the activities evoked by abrupt onsets and orientation contrasts were measured and analyzed. As shown in previous studies, the C1 could start as early as 40–50 ms after stimuli onset (Clark et al., 1995; Di Russo et al., 2003). To avoid possible overlapping from other early ERP components (such as early P1, Ding et al., 2014), the mean amplitudes of the C1 components elicited by abrupt onsets were measured and analyzed at the occipital site POz in the interval of 50–70 ms after stimulus onset. ERPs of orientation contrasts were calculated by subtracting the ERPs of homogeneous stimuli from those of UVF contrast stimuli and those of LVF contrast stimuli. The difference waves were termed as UVF contrast effect (i.e., UVF contrast stimuli − homogeneous stimuli) and LVF contrast effect (i.e., LVF contrast stimuli − homogeneous stimuli), respectively. To examine whether orientation contrast effects occur as early as the C1 component, the UVF contrast effect and the LVF contrast effect were also measured in the 50–70 ms interval at site POz. One sample *t*-tests were performed to examine whether there is a significant C1 effect for the UVF onsets, the LVF onsets, the UVF contrasts and the LVF contrasts, respectively.

To determine the onset time of the UVF/LVF contrast effect, one-sample *t*-tests were carried out with a sliding time window of 20 ms and steps of 2 ms at each posterior scalp site to compare the mean amplitude with zero. The onset time of the contrast effect was defined as the midpoint of the first window at which the activity was significant at the 0.05 level for five or more consecutive windows (Molholm et al., 2002; Kelly et al., 2008).

In addition, a jackknife-based procedure (Miller et al., 1998) was adopted to statistically compare the peak latencies of the earliest contrast effect and the C1 evoked by abrupt onsets at the same locations.

### Result

#### Behavior

Hit rates (Contrast blocks: 87.6 ± 5.6%, Onset blocks: 89.1 ± 6.1%, *t*_(23)_ = 1.125, *p* = 0.272) and RTs (Contrast blocks: 478 ± 9 ms, Onset blocks: 491 ± 12 ms, *t*_(23)_ = 1.960, *p* = 0.062) did not significantly differ between the Contrast and Onset blocks, indicating that the attentional states of participants were similar in these two types of tasks.

#### ERPs

**Figure 2:**
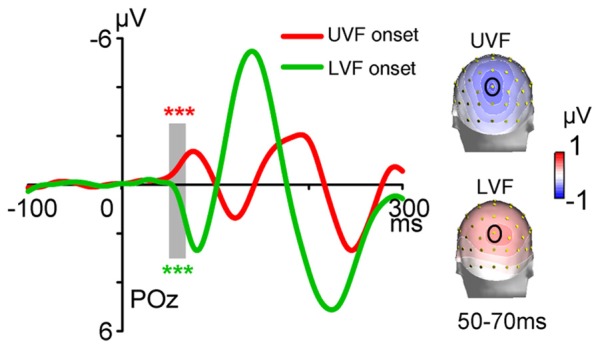
Waveforms and topographies of event-related potentials (ERPs) in Onset blocks of Experiment 1. Waveforms were shown at posterior midline site POz. The C1 evoked by abrupt onsets was negative for stimuli presented in the UVF (red line) and positive for stimuli presented in the LVF (green line). Gray bar indicates the interval of 50–70 ms, and topographies of this interval were shown on the right. Site POz was highlighted by a black circle on topographies. ***p < 0.001.

The ERPs of onset stimuli in Onset blocks are shown in Figure 2. The C1 evoked by abrupt onsets was negative for the UVF and was positive for the LVF over central occipital areas, with maximum amplitudes at POz site and peak latencies at about 80 ms. The polarity, topography and latency all resembled the C1 component reported in previous studies (Di Russo et al., 2003; Rauss et al., 2009; Ding et al., 2014). Further *t*-tests revealed that, during the initial time window of a typical C1 component (i.e., 50–70 ms), the C1 evoked by abrupt onsets was highly significant for both the UVF (site POz: −0.642 ± 0.158 μV, *t*_(23)_ = −4.054, *p* < 0.001; Figure 2, red) and the LVF (0.406 ± 0.081 μV, *t*_(23)_ = 4.988, *p* < 0.001; Figure 2, blue).

**Figure 3:**
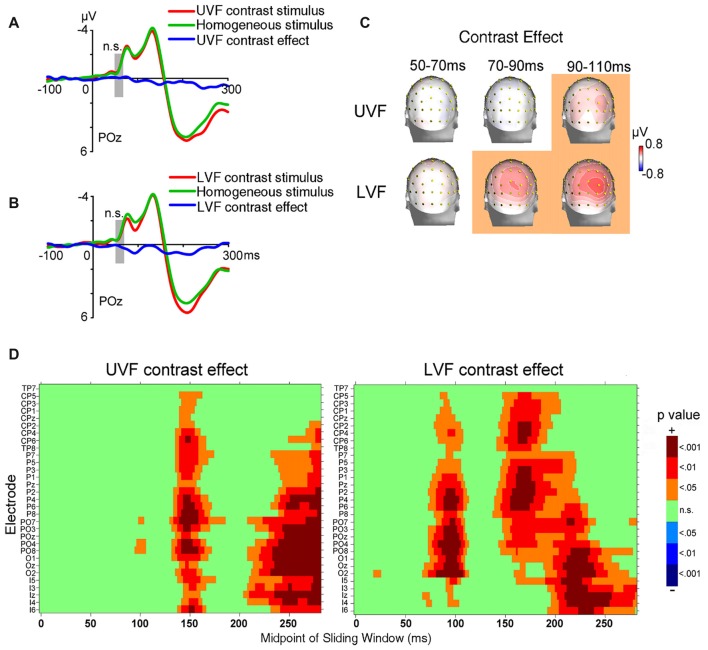
Waveforms and topographies of ERPs in Contrast blocks of Experiment 1. **(A)** The waveforms of ERPs evoked by UVF contrast stimuli, homogeneous stimuli and their difference (i.e., UVF contrast effect). **(B)** The waveforms of ERPs evoked by LVF contrast stimuli, homogeneous stimuli and their difference (i.e., LVF contrast effect). Waveforms were shown at occipital site POz. Gray bars indicate the interval of 50–70 ms. **(C)** Topographies of the UVF contrast effect and LVF contrast effect. Topographies at interval 50–70 ms, 70–90 ms and 90–110 ms were shown. The LVF contrast effect was not significant at 50–70 ms, but was significant at the other two intervals at central posterior sites. The UVF contrast effect was significant only at interval 90–110 ms, with its maximum at lateral occipital sites. The orange background of topographies indicated that significant activities were found at the interval. **(D)** Statistical significance of the UVF and LVF contrast effects at posterior sites. One-sample *t*-tests were carried out with a sliding window of 20 ms and steps of 2 ms at each posterior site to compare the waveform amplitude to zero. *X-axis* in the plots represents the midpoint of sliding window. For example, 100 ms represents the 90–110 ms time window. n.s., not significant.

The ERPs of homogeneous and heterogeneous texture stimuli and their difference waves in Contrast blocks were shown in Figures 3A,B. Both homogeneous and heterogeneous texture stimuli evoked ERPs in posterior occipital areas shortly after stimulus onset (50–70 ms, POz: −0.848 ± 0.187 μV, *t*_(23)_ = −4.538, *p* < 0.001; averaged across UVF contrast stimuli, LVF contrast stimuli, and homogeneous stimuli). However, neither the UVF contrast effect (i.e., difference wave of UVF contrast stimuli and homogeneous stimuli) nor the LVF contrast effect (i.e., difference wave of LVF contrast stimuli and homogeneous stimuli) reached significance during the initial C1 interval (50–70 ms, POz: UVF: −0.088 ± 0.094 μV, *t*_(23)_ = −0.945, *p* = 0.354; Figures 3A,C; LVF: 0.111 ± 0.147 μV, *t*_(23)_ = 0.753, *p* = 0.459; Figures 3B,C).

To reveal the time course and scalp distribution of early activities induced by orientation contrasts, one-sample *t*-tests with sliding windows were used to examine the UVF contrast effect and the LVF contrast effect, respectively (Figure 3D). The earliest significant activities at posterior scalp sites started within 100 ms after stimulus onset for both the UVF and LVF, although latencies and scalp distributions differed: the earliest UVF contrast effect started at 95 ms at occipital site PO8 (0.249 ± 0.115 μV, *t*_(23)_ = 2.136, *p* = 0.044), and reached maximum at around 100 ms at site PO4 (0.580 ± 0.180 μV, *t*_(23)_ = 2.275, *p* = 0.032). By contrast, the earliest LVF contrast effect started at posterior sites at 68 ms (Iz, 0.141 ± 0.066 μV, *t*_(23)_ = 2.136, *p* = 0.044; O2, 0.225 ± 0.104 μV, *t*_(23)_ = 2.163, *p* = 0.041) and reached maximum at around 90 ms at midline occipital site POz (0.512 ± 0.723 μV, *t*_(23)_ = 3.920, *p* < 0.001).

A jackknife-based procedure with a maximum amplitude criterion (Miller et al., 1998) was adopted to compare the latencies of the earliest contrast effect (Figure 3) and the C1 evoked by abrupt onsets presented at the same locations of orientation contrast (Figure 2). We examined the peak latencies of contrast effects and C1s at the sites with maximum amplitudes. The latencies of the early contrast effects were significantly longer than the latencies of C1 in both the UVF (UVF contrast effect at site PO4: 97 ms; C1 at site POz: 77 ms; *t*_(23)_ = 61.575, *p* < 0.001) and LVF (LVF contrast effect at site POz: 93 ms; C1 at site POz: 81 ms; *t*_(23)_ = 70.483, *p* < 0.001), showing a delay of nearly 20 ms in peak latency in the UVF, and a delay of approximate 12 ms in the LVF.

Taken together, these results indicated that, although the early activities related to automatic processing of orientation contrast occurred shortly after (within 100 ms) stimulus onset at occipital sites for both the UVF and LVF, they were still later than the C1 components evoked by abrupt onset stimuli presented at the same locations.

## Experiment 2

In Experiment 1, we revealed that the earliest activities evoked by task-irrelevant orientation contrasts have longer latencies than the C1 components evoked by abrupt onset stimuli presented at the same locations. The delay of activities supports the idea that automatic processing of orientation contrast occurs later than the initial feedforward stage in human V1. Even though the orientation contrasts in Experiment 1 were task-irrelevant and were presented outside the focus of spatial attention, participants might be still aware of these highly salient stimuli (Braun and Sagi, 1990). As shown in recent studies, salient irrelevant stimuli could be actively suppressed due to the awareness of them (Tsushima et al., 2006), and top-down processing in perception history may modulate bottom-up processing of current stimuli (Awh et al., 2012; Qu et al., 2017). One may argue that the orientation contrasts might be actively suppressed due to awareness of them in previous trials, resulting in no obvious orientation contrast effect in the C1 time interval in Experiment 1. To rule out this possibility, we designed Experiment 2, in which the presentation of orientation contrast was immediately followed by backward masks to block participants’ awareness. We then examined whether any earlier contrast effect could be observed.

### Method

#### Participants

Twenty-four healthy young volunteers (seven males, mean age = 20.3 years, range = 19–22 years) took part in Experiment 2. All participants had normal or corrected-to-normal vision and were right-handed. All volunteers were compensated for their participation with payment or with credit hours fulfilling a course requirement. Written informed consent was obtained from each participant before the experiment.

#### Stimuli and Task

Stimuli and procedure were similar to those used in Contrast blocks in Experiment 1 except for the following differences. The presentation of texture stimuli was shortly followed by backward masking stimuli. Masking stimuli (Figure 1C) were constructed by superimposing vertical and horizontal line segments upon one another, resulting in a 19 × 19 array of cross elements (13 cd/m^2^). As shown in Figure 1D, in each trial, the texture stimulus was presented for 33 ms, followed immediately by a masking stimulus (67 ms), and then a blank interval (1,300–1,700 ms).

During EEG recording, participants were required to perform a same central luminance detection task as in Experiment 1. The experiment consisted of nine blocks of 192 trials, resulting in a total of 1,728 trials. The homogenous stimuli, UVF contrast stimuli and LVF contrast stimuli were presented randomly with equal probabilities. Thus, same to Contrast blocks in Experiment 1, there were 576 trials for each type of stimulus.

EEG recording was followed by an awareness test, in which the visibility of orientation contrast was examined. The trial sequence of the awareness test was the same to that used in the EEG recording session. Participants were required to fixate at the central fixation point and to press a predefined key when seeing any peripheral orientation contrast. A total of 96 trials were presented. There were 48 homogeneous stimuli, 24 UVF contrast stimuli and 24 LVF contrast stimuli, respectively.

#### EEG Recordings

The EEG recordings in Experiment 2 were the same as described in Experiment 1. After artifact rejection, about 360 trials were left for each type of stimulus (homogeneous stimulus, UVF contrast stimulus and LVF contrast stimulus).

#### Statistical Analyses

In the central luminance detection task, hit rates and RTs of correct hit responses were calculated. Correct hits were defined as responses from 200 ms to 1200 ms after target onsets as in Experiment 1. Independent sample *t*-tests were used to compare the behavioral performance (i.e., hit rates and RTs) between Experiment 2 and Contrast blocks of Experiment 1. In the awareness test, we measured the accuracy on detection of orientation contrast.

ERP mean amplitudes of the UVF contrast effect and LVF contrast effect were measured and analyzed, respectively. As in Experiment 1, the UVF contrast effect and LVF contrast effect were first measured at POz site at the interval of 50–70 ms after stimulus onset. This time window was used to examine whether any contrast effect occurs in the early C1 interval when backward masking was adopted. In addition, one-sample *t*-tests with sliding windows were carried out to determine the onset time of contrast effect, and a jackknife-based procedure (Miller et al., 1998) was used to examine the peak latency of the earliest contrast effect in posterior area.

### Result

#### Behavior

In Experiment 2, the hit rate and RT of the central luminance detection task were 87.7 ± 0.7% and 461 ± 12 ms, respectively. Both were well matched to those of Contrast blocks in Experiment 1 (hit rates: 87.6 ± 5.6%; RT: 478 ± 9 ms), and no significant difference between experiments was found (hit rate: *t*_(46)_ = 0.150, *p* = 0.882; RT: *t*_(46)_ = 1.813, *p* = 0.076).

Awareness test was conducted after the EEG session to verify the validity of backward masking. The average accuracy (58.89 ± 2.69%) of the test was close to random guessing (50%), suggesting that the awareness of orientation contrast with backward masking was very low even when they were targets. Concerning that the orientation contrasts were totally task-irrelevant in the central luminance detection task, participants should have little awareness of the orientation contrasts in the EEG session. These results indicated that the manipulation of awareness blocking with backward masks was successful.

#### ERPs

**Figure 4:**
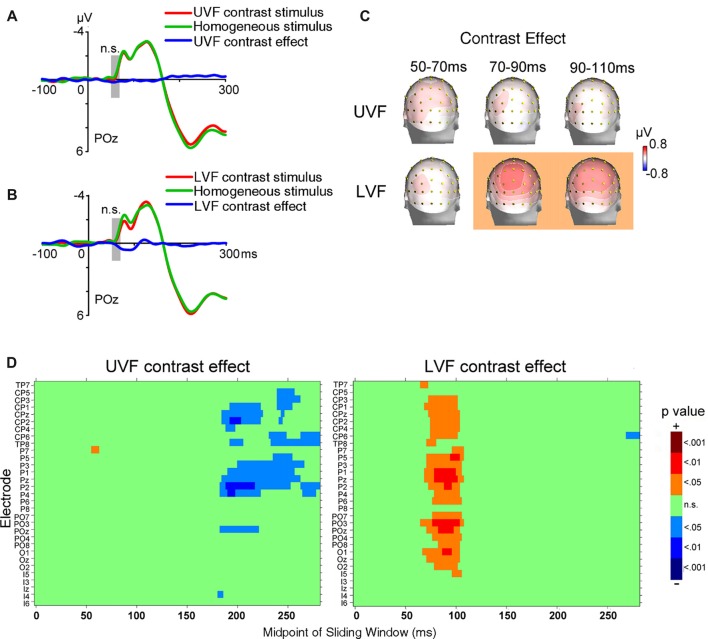
ERP results of Experiment 2. **(A)** The waveforms of ERPs evoked by UVF contrast stimuli, homogeneous stimuli and their difference (i.e., UVF contrast effect). **(B)** The waveforms of ERPs evoked by LVF contrast stimuli, homogeneous stimuli and their difference (i.e., LVF contrast effect). Waveforms were shown at posterior site POz. Gray bars indicate the interval of 50–70 ms. **(C)** Topographies of the UVF contrast effect and the LVF contrast effect. Topographies at interval 50–70 ms, 70–90 ms and 90–110 ms were shown. The LVF contrast effect was not significant at 50–70 ms, but was significant at the other two intervals at central posterior sites. The UVF contrast effect was not significant at any of these intervals. The orange background of topographies indicated that significant activities were found at the interval. **(D)** Statistical significance of the UVF and LVF contrast effects at posterior sites. One-sample *t*-tests were carried out with a sliding window of 20 ms and steps of 2 ms at each posterior site to compare the waveform amplitude to zero. n.s., not significant.

ERPs evoked by texture stimuli were shown in Figures 4A,B. As in Experiment 1, electrophysiological activities related to processing orientation contrast with backward masking were analyzed at the initial C1 time window (50–70 ms after stimulus onset) at central occipital sites. Again, no significant UVF contrast effect (POz, 0.178 ± 0.158 μV, *t*_(23)_ = 1.126, *p* = 0.272) or LVF contrast effect (POz, 0.102 ± 0.136 μV, *t*_(23)_ = 0.750, *p* = 0.461) was found in this interval. In other words, even when the orientation contrasts were of very low visibility, they could not be automatically processed as early as the typical C1.

As shown in Figures 4C,D, when backward masking was used, the earliest significant LVF contrast effect in posterior areas started at 66 ms (PO3, 0.294 ± 0.138 μV, *t*_(23)_ = 2.127, *p* = 0.044) and reached its maximum at 88 ms at midline site POz (0.523 ± 0.848 μV, *t*_(23)_ = 2.737, *p* = 0.012). As revealed by jackknife-based procedure with a maximum amplitude criterion (Miller et al., 1998), the latency of the early LVF contrast effect (site POz: 88 ms) was significantly longer than the C1 evoked by abrupt onset stimuli in Experiment 1 (site POz: 81 ms; *t*_(46)_ = 5.898, *p* < 0.001). By contrast, no significant UVF contrast effect was found within 150 ms after stimulus onset.

In summary, even when awareness of orientation contrast was greatly weakened, we did not found any significant contrast effect in the early time window of the C1. Furthermore, the LVF contrast effect started within 100 ms after stimulus onset, and the latencies and spatial-temporal distributions of the early effect were similar in both the masked and unmasked conditions (Figures 3C, 4C). However, no significant early UVF contrast effect was found in the masked condition.

There are some possible explanations for the difference between visual hemifields. First, since the early activity evoked by orientation contrast is weaker in the UVF relative to in the LVF, it could be more vulnerable to noise. Second, the early UVF effect started later than the early LVF contrast effect in the unmasked condition; as a result, the early UVF contrast effect was more likely to be disrupted by the onset of mask stimuli.

### Further Analysis

#### Onset Latencies of Early Activities

To reveal difference of onset latencies between the C1 components and the orientation contrast effects, we further examined the onset of the C1 in Experiment 1 with moving window *t*-tests. However, the original C1 wave could be vulnerable to the influence of overlapping ERPs of the previous stimulus, which could lead to inaccuracies in the analysis of C1 onset latencies (Qu and Ding, 2018). To remove the possible impact of overlapping components, ERPs of LVF stimuli were subtracted from those of UVF stimuli. The C1 component has reversed polarities for stimuli in the UVF and LVF, while such polarity reversal is absent for most other components. Thus, the UVF-minus-LVF difference wave could eliminate the overlaps from other components and lead to larger C1 and cleaner baseline (Qu and Ding, 2018). According to a previous study (Miller et al., 2015), the subtraction accentuates activities arising from low-level visual areas (e.g., V1–V3) and cancels out activities that do not differ between the UVF and LVF.

**Figure 5:**
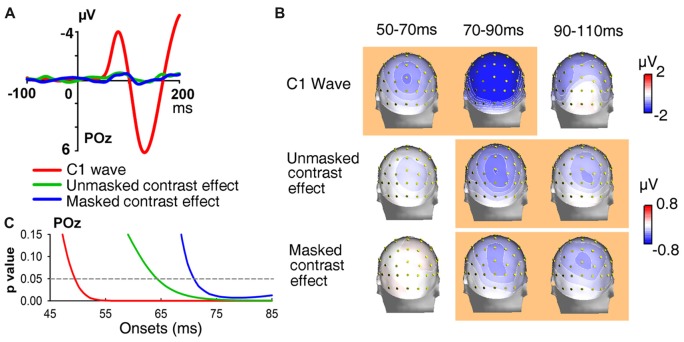
Waveforms, topographies and statistical significance of UVF-minus-LVF difference waves. **(A)** Waveforms of UVF-minus-LVF C1 wave (Experiment 1, red line), as well as UVF-minus-LVF contrast effect in the unmasked condition (Experiment 1, green line) and the masked condition (Experiment 2, blue line). Waveforms were shown at posterior midline site POz. **(B)** Topographies of UVF-minus-LVF C1 wave, UVF-minus-LVF contrast effect in the unmasked condition (Experiment 1) and the masked condition (Experiment 2) at interval 50–70 ms, 70–90 ms and 90–110 ms. The orange background of topographies indicated that significant activities were found at site POz at the interval. The C1 wave of abrupt onset stimuli was significant as early as 50–70 ms, while the UVF-minus-LVF contrast effects were significant at later intervals in both the unmasked and masked conditions. **(C)** Statistical significance of UVF-minus-LVF C1 wave in Experiment 1, and UVF-minus-LVF contrast effect in the unmasked condition (Experiment 1) and the masked condition (Experiment 2) at posterior midline site POz. The onsets of significant activities evoked by both unmasked (green line) and masked orientation contrasts (blue line) were later than that of the C1 wave (red line).

The initial UVF-minus-LVF difference of ERPs evoked by abrupt onset stimuli was a negative C1 over central occipital areas, with its maximum amplitude at site POz at about 80 ms (Figure 5A). As reveled by moving window *t*-tests, the UVF-minus-LVF C1 wave started at occipital site POz at 50 ms (−0.255 ± 0.118 μV, *t*_(23)_ = 2.311, *p* = 0.030, Figure 5C). To compare electrophysiological activities evoked by abrupt onsets and orientation contrasts directly, the UVF-minus-LVF difference of contrast effects was also analyzed with moving window *t*-tests. The UVF-minus-LVF contrast effect reached its maximum at site POz at around 90 ms in both the unmasked and masked conditions (Figure 5A). In the unmasked condition (Experiment 1), a significant UVF-minus-LVF contrast effect occurred at 66 ms at multiple occipital sites (POz, PO4, Oz, O2, Iz, *t*’s_(23)_ < −2.266, *p*’s < 0.033; Figure 5C), which was delayed relative to the onset of C1 wave for about 16 ms. When backward masking was adopted (Experiment 2), significant activities of UVF-minus-LVF contrast effect did not appear at posterior sites until 72 ms (POz, Oz, O1, O2, *t*’s_(23)_ < −2.099, *p*’s < 0.047; Figure 5C).

In addition, as shown in Figure 5B, at the interval of 90–110 ms, the UVF-minus-LVF C1 was not significant any more (POz, −1.084 ± 0.757 μV, *t*_(23)_ = 1.431, *p* = 0.166), while significant UVF-minus-LVF contrast effects still existed in both the unmasked (POz, −0.366 ± 0.121 μV, *t*_(23)_ = 3.030, *p* = 0.006) and masked conditions (POz, −0.400 ± 0.194 μV, *t*_(23)_ = −2.061, *p* = 0.050). Taken together, these findings suggested that both the onset and the offset of the earliest activities evoked by orientation contrasts were later than the C1 components evoked by abrupt onset stimuli.

#### The Influence of Backward Masking

**Figure 6:**
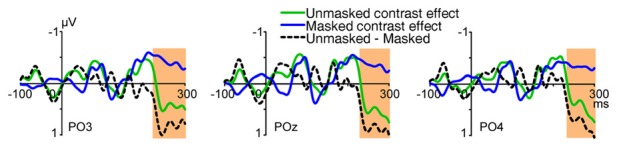
Waveforms of UVF-minus-LVF contrast effects in the unmasked (Experiment 1) and masked (Experiment 2) conditions, and the difference wave (unmasked-minus-masked). Waveforms were shown at posterior sites PO3, POz and PO4. As revealed by independent sample *t*-tests with sliding windows, the difference between the masked and unmasked conditions was not significant at posterior sites until 240–260 ms after stimulus onset. The orange shades indicate the interval where the difference was significant (*p*’s < 0.05).

To examine the impact of backward masking to activities related to processing of orientation contrast, independent sample *t*-tests with sliding windows were used to analyze the difference of contrast effects in the unmasked condition (Experiment 1) and the masked condition (Experiment 2). Results showed that, for the early contrast effects at posterior sites, no significant difference was found between the unmasked and masked conditions (Figure 5B, e.g., POz, PO3, PO4, Oz, O1, O2, 70–90 ms, *t*’s_(46)_ < 0.868, *p*’s > 0.390, 90–110 ms, *t*’s_(46)_ < 0.182, *p*’s > 0.856). Actually, significant influence of backward masking did not appear until around 250 ms after stimulus onset, with more positive deflection in the unmasked condition (e.g., POz, PO3, PO4, Oz, O1, 240–260 ms, *t*’s_(46)_ > 2.489, *p*’s < 0.017, Figure 6). These findings suggested that backward masking did not influence the early orientation effect, but modulated activities at later processing stages. Our results are consistent with a previous study, in which backward masking interrupted ERPs induced by orientation contrasts at longer latencies, while leaving earlier activities intact (Fahrenfort et al., 2007). Different from that study where the orientation contrasts were always targets, in the present study the orientation contrasts were task-irrelevant and outside the focus of top-down spatial attention.

#### Source Localization

To obtain information about the possible sources of the earliest contrast effect in posterior areas, neural generators were estimated from the grand-averaged voltage topographies by distributed linear inverse solutions based on a local autoregressive average (LAURA; Grave de Peralta Menendez et al., 2004). The current implementation of LAURA was generated with a locally spherical head model with anatomical constraints (LSMAC model; Brunet et al., 2010). The solution space included 5,004 nodes equally distributed within the gray matter of the average template brain of the Montreal Neurological Institute (MNI152). No *a priori* assumptions were made regarding the number or location of active sources. The time window for estimating the sources was 70–90 ms after stimulus onset.

**Figure 7:**
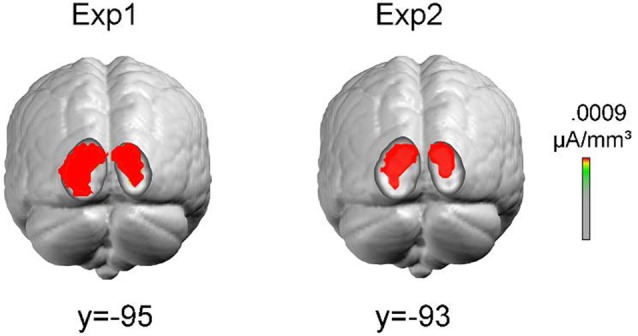
Results of the distributed inverse solution analyses (local autoregressive average, LAURA) used to estimate the neural cortical source of LVF contrast effects. Sources were estimated over the time interval 70–90 ms. Colored areas represent sources with maximal estimated intensity.

As the LVF contrast effect was the earliest activity related to orientation contrast processing, and was relatively stable across two experiments, sources were estimated over the time interval 70–90 ms only for the LVF contrast effect. Sources estimates were calculated on the grand averages in Experiment 1 and Experiment 2, respectively. As shown in Figure 7, the sources were located at similar cortical locations (corresponding to Middle Occipital Gyrus, BA18. Experiment 1: maximum at Talairach coordinates (15, −95, 13); Experiment 2: maximum at Talairach coordinates (15, −93, 11)).

## Experiment 3

In both Experiment 1 and 2, no reliable contrast effect was found on the C1 component and the earliest contrast effect appeared 10–20 ms later than the C1 elicited by the abrupt onsets at same locations. However, one may argue that early contrast effects might depend on where the overall texture stimuli are presented. Both Experiment 1 and 2 used texture stimuli covering both the UVFs and LVFs, which might cancel out any contrast effect on the C1 component. To test this possibility, in Experiment 3, texture stimuli were presented in only the UVF or LVF in any given trial, but orientation contrasts were embedded in the same locations as in Experiment 1 and 2. With such a design, we expect to find typical C1 components with retinotopic polarity reversal elicited by the UVF and LVF texture stimuli. Then we examined whether any contrast effect would be found on the C1 component.

### Method

#### Participants

Twenty-four healthy young volunteers (14 males, mean age = 21.1 years, range = 18–28 years) took part in Experiment 3. All participants had normal or corrected-to-normal vision and were right-handed. All volunteers were compensated for their participation with payment or with credit hours fulfilling a course requirement. Written informed consent was obtained from each participant before the experiment.

#### Stimuli and Task

**Figure 8:**
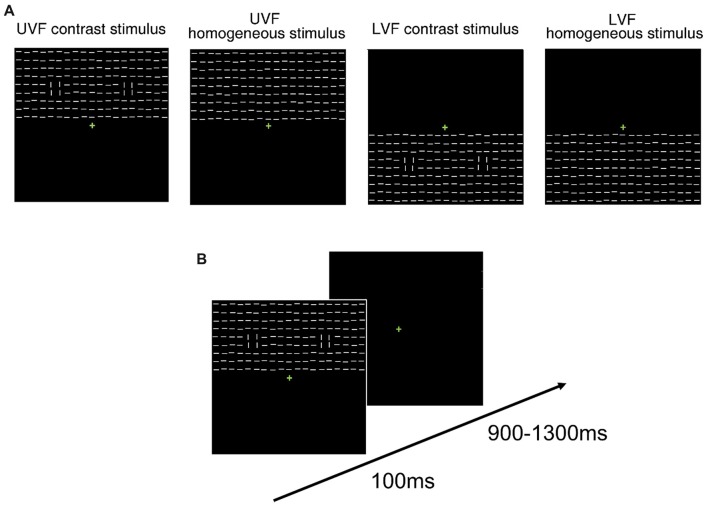
Schematic presentation of stimuli and trial procedure of Experiment 3. **(A)** Schematic presentation of the task-irrelevant textures presented in Experiment 3. Homogeneous and heterogeneous texture stimuli embedded with orientation contrasts were presented in either the UVF or LVF in a trial. For each type of stimulus, 50% were made of horizontal background elements (as shown in the figure), and the other 50% were made of vertical background elements. **(B)** Schematic presentation of the trial procedure in Experiment 3. Participants performed a luminance detection task at fixation while the texture arrays were presented as task-irrelevant stimuli.

Stimuli and procedure were similar to those used in Contrast blocks in Experiment 1 except for the following differences. Each texture stimulus consisted of a 19 × 9 array of line segments (21.7° × 10.0°, Figure 8), and was presented in either the UVF or LVF in a trial. There were four types of texture stimuli: UVF contrast stimulus, UVF homogeneous stimulus, LVF contrast stimulus and LVF homogeneous stimulus (Figure 8A). The four types of stimuli were presented randomly in a block with equal probabilities. There were nine blocks of 256 trials, resulting in a total of 2,304 trials. Thus, same to Experiment 1 and Experiment 2, there were 576 trials for each type of stimulus.

#### EEG Recordings

A NeuroScan EEG/ERP acquisition system, with SynAmps RT amplifier and SCAN software, was used in EEG recording. EEGLAB^1^ was used in offline EEG data processing. The scalp EEG was recorded from an array of 64 electrodes (including the following sites: FP1, FPZ, FP2, AF7, AF3, AF4, AF8, F7, F3, FZ, F4, F8, FT7, FC5, FC3, FC1, FCZ, FC2, FC4, FC6, FT8, T7, C5, C3, C1, CZ, C2, C4, C6, T8, M2, TP7, CP5, CP3, CP1, CPZ, CP2, CP4, CP6, TP8, P9, P7, P5, P3, P1, PZ, P2, P4, P6, P8, P10, PO7, PO3, POZ, PO4, PO8, O1, OZ, O2, I5, I3, IZ, I4, I6 from the 10/20 system). The horizontal and vertical EOG was recorded as well. The EEG was recorded with a reference to right mastoid on-line, and was then algebraically re-referenced to the average of the left and right mastoid. Electrode impedance was kept to less than 5 kΩ. The EEG analog signal was digitized at a 500-Hz sampling rate. A digital low-pass filter with a half-amplitude cutoff frequency of 100 Hz, and a decoupling single pole RC high-pass filter (0.05 Hz, −6 dB/octave/pole) were applied at the time of recording. During the offline analysis, a Blackman windowed sinc FIR filter was used for low-pass filtering on the continuous EEG data, with a half-amplitude cut-off frequency of 40-Hz and transition bandwidth of 20-Hz. Then the epoch was extracted, including 200 ms of pre-stimulus and 600 ms of post-stimulus. The trials contaminated by eye blinks, eye movement or muscle potentials exceeding ±70 μV at any electrode were excluded before averaging, as were data surrounding a button press (−650 to +650 ms). After artifact rejection, about 400 trials were left for each type of stimulus (i.e., UVF homogeneous stimulus, UVF contrast stimulus, LVF homogeneous stimulus, LVF contrast stimulus). The baseline for ERP measurements was the mean voltage over the 100 ms pre-stimulus to stimulus onset, and the average waveforms were corrected by subtracting the mean voltage during this interval.

#### Statistical Analyses

In the central luminance detection task, hit rates and RTs of correct hit responses were calculated. Correct hits were defined as responses from 200 ms to 1200 ms after target onsets as in Experiment 1 and Experiment 2. A one-way ANOVA was used to compare the performance (hit rates and RTs) in Experiment 3, Experiment 2, and Contrast blocks of Experiment 1. Greenhouse-Geisser correction was used where appropriate.

ERP mean amplitudes of the activities evoked by the whole texture stimuli and those evoked by orientation contrasts were measured and analyzed. Similar to Experiment 1, the mean amplitudes of the C1 evoked by UVF stimuli (i.e., average of UVF homogeneous stimuli and UVF contrast stimuli) and LVF stimuli (i.e., average of LVF homogeneous stimuli and LVF contrast stimuli) were measured at the occipital site POz in the interval of 50–70 ms after stimulus onset. ERPs of orientation contrasts were calculated by subtracting the ERPs of homogeneous stimuli from those of contrast stimuli presented in the same hemifield. The difference waves were termed as UVF contrast effect (i.e., UVF contrast stimuli − UVF homogeneous stimuli) and LVF contrast effect (i.e., LVF contrast stimuli − LVF homogeneous stimuli), respectively. As in Experiment 1 and Experiment 2, the UVF contrast effect and the LVF contrast effect were first measured at POz at the interval of 50–70 ms after stimulus onset. This time window was used to examine whether any contrast effect occurs in the early C1 time window. In addition, one-sample *t*-tests with sliding windows were carried out to examine the onset times of the UVF/LVF contrast effect, as well as the onset times of the UVF-minus-LVF C1 component and UVF-minus-LVF contrast effect. A jackknife-based procedure (Miller et al., 1998) was used to statically compare the peak latencies of the earliest contrast effect and the C1 evoked by the texture stimulus presented in the same hemifield.

### Result

#### Behavior

In Experiment 3, the hit rate and RT of the central luminance detection task were 84.0 ± 1.4% and 492 ± 10 ms, respectively. No significant difference in hit rates (*F*_(2, 69)_ = 0.870, *p* = 0.424) or RTs (*F*_(2, 69)_ = 2.510, *p* = 0.089) was found across Experiment 3, Experiment 2, and Contrast blocks in Experiment 1.

#### ERPs

**Figure 9:**
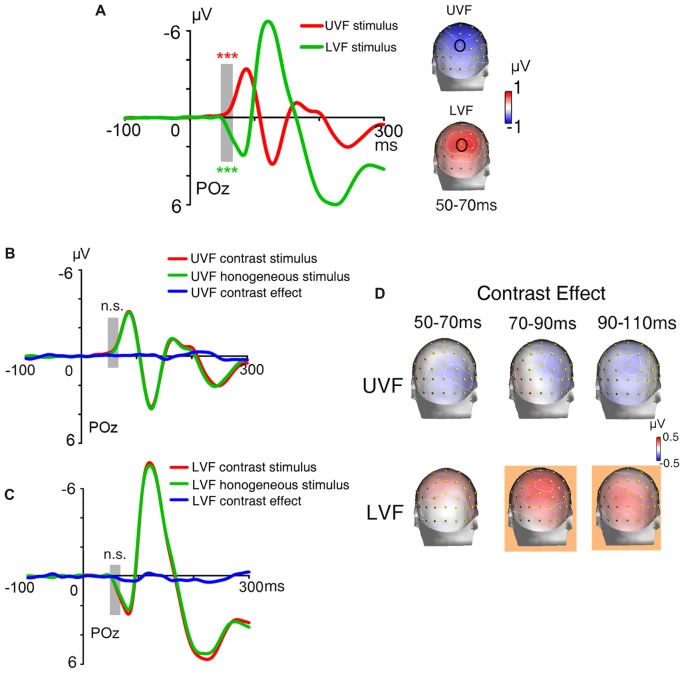
Waveforms and topographies of ERPs in Experiment 3. **(A)** Waveforms and topographies of ERPs evoked by the whole textures. The waveform of the UVF stimuli was averaged across UVF homogeneous stimuli and UVF contrast stimuli, and the waveform of LVF stimuli was averaged across LVF homogeneous stimuli and LVF contrast stimuli. The C1 was negative for stimuli presented in the UVF (red line) and positive for stimuli presented in the LVF (green line). Gray bar indicates the interval of 50–70 ms, and topographies of this interval were shown on the right. Site POz was highlighted by a black circle on topographies. **(B)** The waveforms of ERPs evoked by UVF contrast stimuli, UVF homogeneous stimuli, and their difference (i.e., UVF contrast effect). **(C)** The waveforms of ERPs evoked by LVF contrast stimuli, LVF homogeneous stimuli, and their difference (i.e., LVF contrast effect). Gray bars indicate the latency of 50–70 ms. **(D)** Topographies of the UVF contrast effect and LVF contrast effect. Topographies at interval 50–70 ms, 70–90 ms and 90–110 ms were shown. The LVF contrast effect was not significant at 50–70 ms, but was significant at 70–90 ms and 90–110 ms. The UVF contrast effect was not significant at any of these intervals. The orange background of topographies indicated that significant activities were found at the interval. ***p < 0.001. n.s., not significant.

ERPs evoked by texture stimuli were shown in Figure 9A. The C1 evoked by the whole texture was negative for the UVF and was positive for the LVF over central occipital areas, with maximum amplitudes at around POz site and peak latencies at about 80 ms. The polarity, topography and latency all resembled the C1 component reported in previous studies (Di Russo et al., 2003; Ding et al., 2014). Both the UVF and LVF stimuli evoked highly significant C1 with reversed polarities during the interval 50–70 ms (site POz, UVF: −0.501 ± 0.105 μV, *t*_(23)_ = −4.749, *p* < 0.001; LVF: 0.631 ± 0.126 μV, *t*_(23)_ = 5.001, *p* < 0.001; Figure 9A).

The ERPs of homogeneous and heterogeneous texture stimuli and their difference waves were shown in Figures 9B,C. The mean amplitudes of the UVF contrast effect (i.e., difference wave of UVF contrast stimuli and UVF homogeneous stimuli) and the LVF contrast effect (i.e., difference wave of LVF contrast stimuli and LVF homogeneous stimuli) were measured at interval 50–70 ms respectively. Results showed that, in contrary to the C1 component, neither the UVF contrast effect (POz, −0.076 ± 0.083 μV, *t*_(23)_ = −0.919, *p* = 0.367; Figures 9B,D) nor the LVF contrast effect (POz, 0.079 ± 0.080 μV, *t*_(23)_ = 0.986, *p* = 0.335; Figures 9C,D) was significant at the 50–70 ms interval.

To measure the time course of early activities induced by orientation contrasts, *t*-tests with sliding windows were used to examine both the UVF and LVF contrast effects. The LVF contrast effect started at 68 ms at midline site POz (0.171 ± 0.074 μV, *t*_(23)_ = 2.315, *p* = 0.030), and reached its maximum at around 92 ms (0.301 ± 0.083 μV, *t*_(23)_ = 3.645, *p* = 0.001). By contrast, no significant UVF contrast effect was found within 150 ms after stimulus onset. A jackknife-based procedure with a maximum amplitude criterion (Miller et al., 1998) was adopted to compare the latencies of the C1 and contrast effect in the LVF. Results showed that the latency of the early LVF contrast effect was significantly longer than that of the C1 in the same visual field (LVF contrast effect at site POz: 94 ms; LVF C1 at site POz: 84 ms; *t*_(23)_ = 14.480, *p* < 0.001), with a delay of approximate 10 ms.

**Figure 10:**
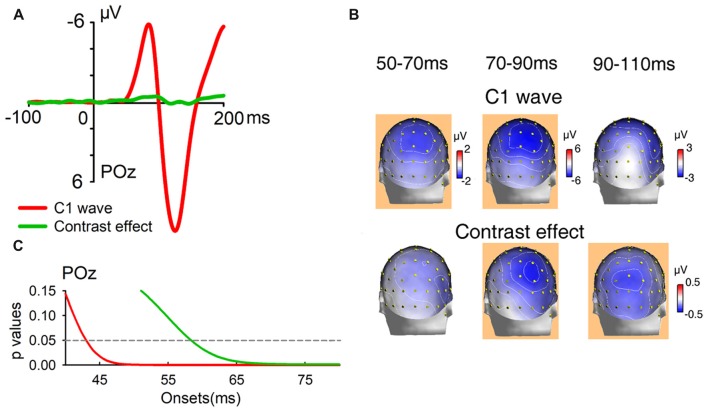
Waveforms, topographies and statistical significance of UVF-minus-LVF difference waves in Experiment 3.** (A)** Waveforms of UVF-minus-LVF C1 wave (red line) and UVF-minus-LVF contrast effect (green line). Waveforms were shown at posterior midline site POz. **(B)** Topographies of UVF-minus-LVF C1 wave and UVF-minus-LVF contrast effect at interval 50–70 ms, 70–90 ms and 90–110 ms. The C1 wave of texture stimuli was significant as early as 50–70 ms, while the UVF-minus-LVF contrast effect was significant at later intervals. The orange background of topographies indicated that significant C1 component or contrast effect was found at site POz at the interval. **(C)** Statistical significance of UVF-minus-LVF C1 wave and UVF-minus-LVF contrast effect at posterior midline site POz. The onset of significant activity evoked by orientation contrast (green line) was later than that of the C1 wave (red line).

As in Experiment 1 and 2, we also measured the UVF-minus-LVF difference waves of the C1 component and the contrast effects. As shown in Figure 10A, the initial UVF-minus-LVF difference of ERPs evoked by the texture stimuli was a negative C1 over central occipital areas, with its maximum amplitude at site POz at about 80 ms. As reveled by moving window *t*-tests, the UVF-minus-LVF C1 wave started at occipital site POz at 50 ms (−0.294 ± 0.128 μV, *t*_(23)_ = −2.298, *p* = 0.031, Figure 10C). By contrast, a significant UVF-minus-LVF contrast effect occurred at 66 ms at occipital site POz (−0.213 ± 0.094 μV, *t*_(23)_ = −2.268, *p* = 0.033; Figure 10C), which was delayed relative to the onset of the C1 for about 16 ms. In addition, as shown in Figure 10B, at a later interval of 90–110 ms, the UVF-minus-LVF C1 became insignificant (POz, 0.366 ± 1.135 μV, *t*_(23)_ = 0.323, *p* = 0.750), while there was still a significant UVF-minus-LVF contrast effect (POz, −0.339 ± 0.111μV, *t*_(23)_ = −3.038, *p* = 0.001). These findings replicated the results of Experiment 1 and 2, suggesting that both the onset and the offset of the earliest activities evoked by orientation contrasts were later than the C1 components evoked by abrupt onset stimuli.

In summary, in Experiment 3, the overall texture stimulus occupies either the UVF or LVF in a given trial, whereas the locations of the embedded orientation contrasts are the same as in Experiment 1 and 2. As predicted, such texture stimuli evoked strong and typical C1 components with polarity reversal for the UVF and LVF. However, no reliable contract effect was found on the C1 component, and the earliest contrast effect appeared 10–20 ms later than the onset of C1 component. Moreover, the early contrast effect was more robust in the LVF than in the UVF. All these results were consistent with the findings in Experiment 1 and 2. Taken together, our results indicate that early effects of orientation contrast are determined by where the contrasts are located rather than where the overall texture stimuli are presented.

## Discussion

The present study investigated the early automatic processing of orientation contrast with experiment settings that minimized the influence of top-down attention and facilitated the observation of early scalp ERPs. In addition, we examined whether the early automatic processing of orientation contrast depends on visual awareness. We found that orientation contrasts evoked significant activities in posterior areas within 100 ms after stimulus onset. This early effect of orientation contrast depended on where the orientation contrasts were embedded in a texture stimulus rather than where the overall texture stimulus was located, and existed even when orientation contrasts were invisible due to backward masking. Nevertheless, even though orientation contrasts were presented in optimal locations for the observation of early scalp ERPs, we still found no evidence that the C1 component could be modulated by orientation contrast.

By examining the ERPs evoked by abrupt onset stimuli presented in the same locations of orientation contrasts, we compared the time courses of the early effect of orientation contrast and the C1 evoked by abrupt onset stimuli directly. First, the C1 evoked by abrupt onset stimuli became significant shortly (i.e., 50–70 ms) after stimulus onset, while an orientation contrast effect did not emerge at the same interval. Second, the peak latency of the early contrast effect in visual cortical areas was significantly longer than that of C1 in both the UVF and LVF. Third, by analyzing the UVF-minus-LVF waves, we found that the orientation contrast effects in both the masked and unmasked conditions started later than the C1 for about 20 ms. In addition, while the C1 components in the UVF and LVF were of reversed polarities, no such polarity reversal was found for the early effects of orientation contrast. Under some certain condition (e.g., Experiment 1), the early orientation contrast effects even showed a similar positive polarity for both hemifields. Taken together, the present study provides convergent evidence that automatic processing of orientation contrast occurs at later stages than the initial feedforward stage in V1. Our findings are in line with previous single unit studies in monkeys (Knierim and van Essen, 1992; Zipser et al., 1996; Nothdurft et al., 1999; Poort et al., 2012), and provide human ERP evidence regarding the time course of the automatic processing of orientation contrast.

Though the automatic processing of orientation contrast did not start during initial feedforward stage in V1, our results suggested the involvement of early visual cortical processing stages. In particular, the delay of the early LVF contrast effect relative to C1 was less than 20 ms, suggesting the involvement of relatively early processing stages. Such reasoning was supported by the topographic distribution of the early contrast effect, which showed its maximum at central posterior sites. Additionally, sources of the early contrast effect were also located in low level visual cortex. Taking all these evidences into consideration, the orientation contrast effect could occur in relatively early processing stages in low level visual areas. Relative to most previous ERP studies that presented orientation contrasts either as targets (Caputo and Casco, 1999; Schlaghecken et al., 2001; Fahrenfort et al., 2007; Straube et al., 2010) or as task-irrelevant stimuli (Schubö et al., 2001; Straube et al., 2010; Guzzon and Casco, 2011), we revealed an orientation contrast effect at a shorter latency. The earlier effect could benefit from stimulus locations that are suitable for the observation of early ERPs. Also, the early effect was revealed with more repetition of stimulus presentation in the current study than in previous studies, which could lead to a higher signal-to-noise ratio.

One possible mechanism underlying the early orientation contrast effect is that it could reflect recurrent activities in V1 (Lamme et al., 1998a; Lamme and Roelfsema, 2000). The recurrent activities might be feedback signals from higher level cortical areas, or signals from horizontal connections within V1. Previous studies found that recurrent modulation from high level cortical areas to low level cortical areas could occur very quickly (Pascual-Leone and Walsh, 2001; Boehler et al., 2008). For example, in the study of Boehler et al. (2008), rapid recurrent modulation from extrastriate areas to V1 occurred only 27 ms after the initial feedforward activities in V1 and only 11 ms after the onset of activities in extrastriate areas. The early contrast effect in the current study is in line with these findings. Nevertheless, we cannot rule out the possibility that the current effect of orientation contrast might involve feedforward activities from V1 to other higher visual areas (Fahrenfort et al., 2007), such as V2 or V3. Even though more evidence is needed to determine the nature of the contrast effect, the current study shows that automatic processing of orientation contrast occurs slightly later than initial feedforward activities in V1.

We also found that the early effects of orientation contrast in the posterior area are asymmetric between the UVF and LVF. Relative to the contrast effect in the UVF, the contrast effect in the LVF started earlier and had larger amplitudes. In addition, the early LVF contrast effect reached its maximum at central posterior sites, whereas the early UVF contrast effect was distributed more bilaterally. These differences suggest that there might be a LVF advantage in automatic processing of orientation contrast. Asymmetries between the UVFs and LVFs were reported in previous studies in many perceptual tasks (He et al., 1996; Rubin et al., 1996; Qu et al., 2006; Pourtois et al., 2008; Rauss et al., 2009; Bombeke et al., 2016). Previous studies also found that contrast sensitivity (Skrandies, 1985) and spatial resolution (Skrandies, 1987) in the LVF is higher than in the UVF. These differences between the UVFs and LVFs could lead to the asymmetries in the current study, especially the latency advantage of LVF. Another possible source of the asymmetries is that orientation contrasts in the UVF and LVF might project to different cortical surfaces in low level visual areas (Clark et al., 1995). The difference in the anatomical projection could also result in difference of ERPs recorded on the scalp. More research is needed to fully clarify these explanations.

In the current study, the early orientation contrast effect was found not only when orientation contrasts were task-irrelevant and presented outside top-down attention focus, but also when awareness of the contrasts was greatly weakened by backward masking. Although automatic processing of orientation contrast which is independent of top-down attention has been revealed in previous ERP studies, the role of visual awareness in early automatic processing was not examined (Schubö et al., 2001; Scholte et al., 2008; Guzzon and Casco, 2011). In some other studies (Fahrenfort et al., 2007; Zhang et al., 2012), visual awareness of orientation contrasts was reduced by backward masking at short latencies, but orientation contrasts were always the targets and were therefore subjected to top-down spatial attention. In such situation, activities related to automatic processing (even those early ones) could be mixed with activities related to top-down influence. Compared with these studies, the current study revealed early orientation contrast effects in the absence of top-down spatial attention as well as visual awareness, suggesting that orientation contrasts could modulate early visual cortical processing independent of both top-down attention and visual awareness.

Although we adopted stimuli and parameters similar to those in Zhang et al. (2012), the findings of the two studies were different. Zhang et al. (2012) found that C1 amplitude was modulated by orientation contrast, which was regarded as evidence of a saliency map organized in V1. Orientation contrasts in their study were always targets, since participants had to discriminate the location of the contrast region. In contrast, in our study, orientation contrasts were task-irrelevant and were far away from the focus of top-down spatial attention, and the C1 component was not modulated by orientation contrast. The difference manipulation of top-down attention to orientation contrasts may account for the discrepancies between the two studies. It is possible that top-down attention is critical for the processing of orientation contrast occurring in initial feedforward stage in V1. Further studies are needed to verify this possibility. In addition, since the time course and cortical sources of the contrast effect were not reported in Zhang et al. (2012), it is still an open question whether it really reflected changes of the C1 component.

In summary, the present study shows that automatic processing of orientation contrast could start rapidly even when the orientation contrasts were totally task irrelevant and were immediately followed by visual masks. These early activities might reflect automatic processing that is independent of top-down attention and visual awareness. The early activities evoked by orientation contrasts in low level visual areas occur later than C1 evoked by abrupt onset stimuli at the same locations, suggesting that automatic processing of orientation contrast does not occur in the initial feedforward processing in V1.

## Author Contributions

YD and ZQ conceived and designed the study. YZ, DL, RD and ZH performed the experiments and analyzed the data under the supervision of YD and ZQ. YZ, YD and ZQ wrote the manuscript.

## Conflict of Interest Statement

The authors declare that the research was conducted in the absence of any commercial or financial relationships that could be construed as a potential conflict of interest.

## Footnotes

^1^http://sccn.ucsd.edu/eeglab/
